# Supplementary material for: Causal Relationship between Aging and Anorexia Nervosa: A White-Matter-Microstructure-Mediated Mendelian Randomization Analysis
Source: Biomedicines. 2024 Aug 16;12(8):1874. doi: 10.3390/biomedicines12081874 (PMC11351342; doi:10.3390/biomedicines12081874)
Supplement: Supplementary file 1 [file biomedicines-12-01874-s001.zip › Supplementary Figure/Figure S8/Leaveoneout plot of MR analysis for TL to ubm-b-1494.pdf]

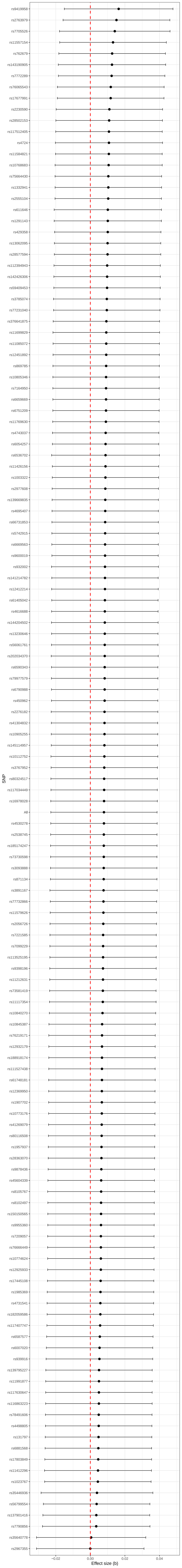
rs9419958
rs2763979
rs7705526
rs11557154
rs762679
rs143190905
rs7772289
rs76065543
rs17677991
rs2230590
rs28502153
rs117512405
rs4724
rs11584821
rs10768683
rs75664430
rs1332941
rs2555104
rs611646
rs1291143
rs429358
rs13062095
rs28577594
rs112394943
rs142426306
rs59409453
rs3785074
rs77231040
rs376641875
rs11699829
rs11085072
rs12451892
rs869785
rs10805346
rs7164950
rs6659669
rs6751209
rs11769630
rs4743037
rs6054257
rs6536702
rs11426156
rs1003322
rs2977608
rs139669835
rs4695407
rs66731853
rs5742915
rs6669563
rs9600019
rs932002
rs141214782
rs12412214
rs61405042
rs4616688
rs144204502
rs13230646
rs56061761
rs202034370
rs6590343
rs79977579
rs6790988
rs450962
rs2276182
rs41304832
rs10905255
rs145114957
rs10112752
rs3767952
rs80324517
rs117034449
rs16978028
A8
rs4530278
rs2538745
rs185174247
rs73730598
rs3093888
rs871134
rs3891167
rs77732866
rs11579626
rs2056726
rs7221585
rs7099229
rs113525195
rs9398196
rs11212631
rs73581419
rs11117354
rs10840270
rs10845387
rs76219171
rs12932179
rs188918174
rs111527438
rs61748181
rs12369950
rs1907702
rs10773176
rs41269079
rs80116508
rs1957937
rs28363070
rs9878436
rs45604339
rs8105767
rs8102497
rs150150565
rs9955360
rs7209057
rs76666449
rs10774624
rs12925933
rs17445108
rs1985369
rs4731541
rs182059586
rs117407747
rs6587577
rs6007020
rs939916
rs139795227
rs11991877
rs117630647
rs116863223
rs78491606
rs4498805
rs131797
rs6881568
rs17803849
rs11412296
rs1023767
rs35446936
rs56799554
rs137901416
rs7790856
rs35640778
rs2967355
SNP
-0.02
0.00
0.02
0.04
Effect size (b)
